# Special Issue “Viral and Host Factors Driving the Emergence and the Evolution of the SARS-CoV-2 and Other Coronaviruses”

**DOI:** 10.3390/v14081705

**Published:** 2022-08-02

**Authors:** Corinne Ronfort

**Affiliations:** 1 Animal Health Department UMR IVPC, French National Research Institute for Agriculture, Food and Environment, University of Lyon I, 69007 Lyon, France; corinne.ronfort@inrae.fr; 2 AIOVA sas, 570 rue de la Chimie, University of Grenoble, 38300 Saint Martin d’Hèyres, France

Two and half years ago, humanity was facing the emergence of the Severe Acute Respiratory Syndrome Coronavirus 2 (SARS-CoV-2), the causal agent of the COVID-19 pandemics that significantly impact public health, society and the global economy. As of 5 July 2022, the virus has officially infected more than 546 million people, killing over 6.3 million globally [https://covid19.who.int (accessed on 5 July 2022).]. However, the World Health Organization (WHO) recently estimated that approximately 15 million people died during the first two years of the COVID-19 pandemic [1].

This emergence is the third in just two decades after the SARS-CoV-1 in 2002 and the MERS in 2012. Four other coronaviruses that are responsible for seasonal colds (HCoV-229E, HCoV-NL63, HCoV-OC43, HCoV-HKU1) have circulated in the human population for decades. Of note, phylogenic, paleo-serological and clinical observations consider the role of a coronavirus in the 1980 Russian influenza pandemics and mark HCoV-OC43 as a plausible candidate [2].

Altogether, these observations strongly suggest that new SARS pandemics are likely to occur in the near future.

This special issue aims to provide data on the viral, host and environmental factors involved in the emergence and evolution of the SARS viruses. Herein, we publish several papers that studied this topic from different angles.

Due to their RNA nature, SARS viruses undergo continuous genetic changes, including mutations, deletion/insertions and recombinations. In the following series of articles, the authors evaluated the viral epidemiology, transmission dynamics and evolution in different populations and regions worldwide.

Capoferri et al. studied the diversity of the virus during the first year of the pandemic (2020 in the U.S.) before the vaccination campaign and explained the capacity of SARS-CoV-2 when mutating in a naïve population [3]. The virus spread to an almost completely immunologically naïve population, and the viral genetic diversity increased over time, tripling in this period.

Hui Min Chan et al. analyzed the SARS-CoV-2 population genetics in different regions across the globe [4]. The authors used a multilocus genotyping tool in combination with population genetic approaches to monitor viral transmission dynamics and evolution globally at two time points (December 2019 to September 2020 and January to March 2021) in different regions worldwide. The authors uncovered contrasting epidemiology and evolution of viral populations within and between the continents.

Gonzales-Puelma et al. reported and analyzed the occurrence and the molecular evolution of a new haplotype (carrying the T307I spike mutation) that expanded and completely replaced the previous lineage within six months in a geographically-isolated population in Chile [5]. This change was associated with a significant increase in infection rates.

By contrast, Limaye et al. analyzed variants circulating in a populous country (India) with densely packed cities during a 17-month period, giving us an interesting account of pan-Indian diversity [6]. The authors detected substantial multiple introductions due to global and interstate travel and showed that variants such as alpha, beta, delta, kappa (that emerged in India), eta, gamma and iot were in circulation at different times points of the study.

All SARS viruses are of animal origin and manifest a high propensity for interspecies transmission. In an interesting review, Islam et al. evaluated the epidemiology and evolutionary dynamics of the coronavirus diversity in humans, animals (companion and livestock) and wildlife, as well as a timeline of the emergence of human coronaviruses, their reservoirs, and their intermediate hosts [7].

Of note, it has recently been shown that 30 to 40% of white-tailed deer (Odocoileus virginianus) in the northeastern United States have been infected with SARS-CoV2. Ostensibly, the virus spilled over from humans, and the deer have spread the infection among themselves [8]. Furthermore, in farmed mink in Denmark, it has been shown that an infection was initiated by humans, followed by the emergence of a mink variant and the transmission from the mink back to humans again (reverse zoonosis) [9].

Thus, there is a growing concern about the animals becoming a viral reservoir, serving as a source of outbreaks and potentially breeding new variants. More knowledge on coronaviruses circulating in animals is crucial to preventing the evolution of SARS viruses and the emergence of new variants.

Finally, an issue is the role played by vaccination as a source of coronavirus evolution and the capacity of coronaviruses for escaping from vaccine-induced immunity. Poultry companies have been using a vaccine against the Infectious Bronchitis Virus (IBV) worldwide for a long time, but the emergence of novel serotypes makes it challenging to control. Flageul et al. showed that vaccinations that induce partial protection can be one of the driving factors of IBV evolution, which undoubtedly contributes to the emergence of new strains [10]. They detected several variants in vaccinated and unvaccinated animals. These populations of viruses did not evolve the same in birds with different IBV immune statuses. These observations raise the issue of the impact of the current vaccines on the genetic evolution of SARS-CoV-2 in humans.

In conclusion, in the face of SARS-CoV-2, a coronavirus with a high capacity to mutate, which circulates in numerous animals with the potential of reverse zoonosis (reinfection of humans) and that can escape from vaccine-induced immunity, humanity urgently needs vaccines that block both infection and transmission and achieve complete protection to avoid genetic drift. This should be performed in a One Health approach.

I sincerely thank the authors, reviewers and the editorial staff members for their contribution to this special issue.
